# Supplementary material for: Predicting visual function by interpreting a neuronal wiring diagram
Source: Nature. 2024 Oct 2;634(8032):113–23. doi: 10.1038/s41586-024-07953-5 (PMC11446822; doi:10.1038/s41586-024-07953-5)
Supplement: Supplementary file 6 — CRF and ERF predictions for individual TmY4 and TmY9 cells. Analogous to Supplementary Data 3, but for TmY target types. Shown are the top four monosynaptic pathways, the strongest pathway passing through each of the top ten intermediary types (ranking from Extended Data Fig. 7), and the trisynaptic pathway Tm1–TmY–Dm3–TmY (see the section entitled Prediction of spatial normalization). [file 41586_2024_7953_MOESM6_ESM.zip › DataS4/TmY9qΓèÑ/720575940620260372.pdf]

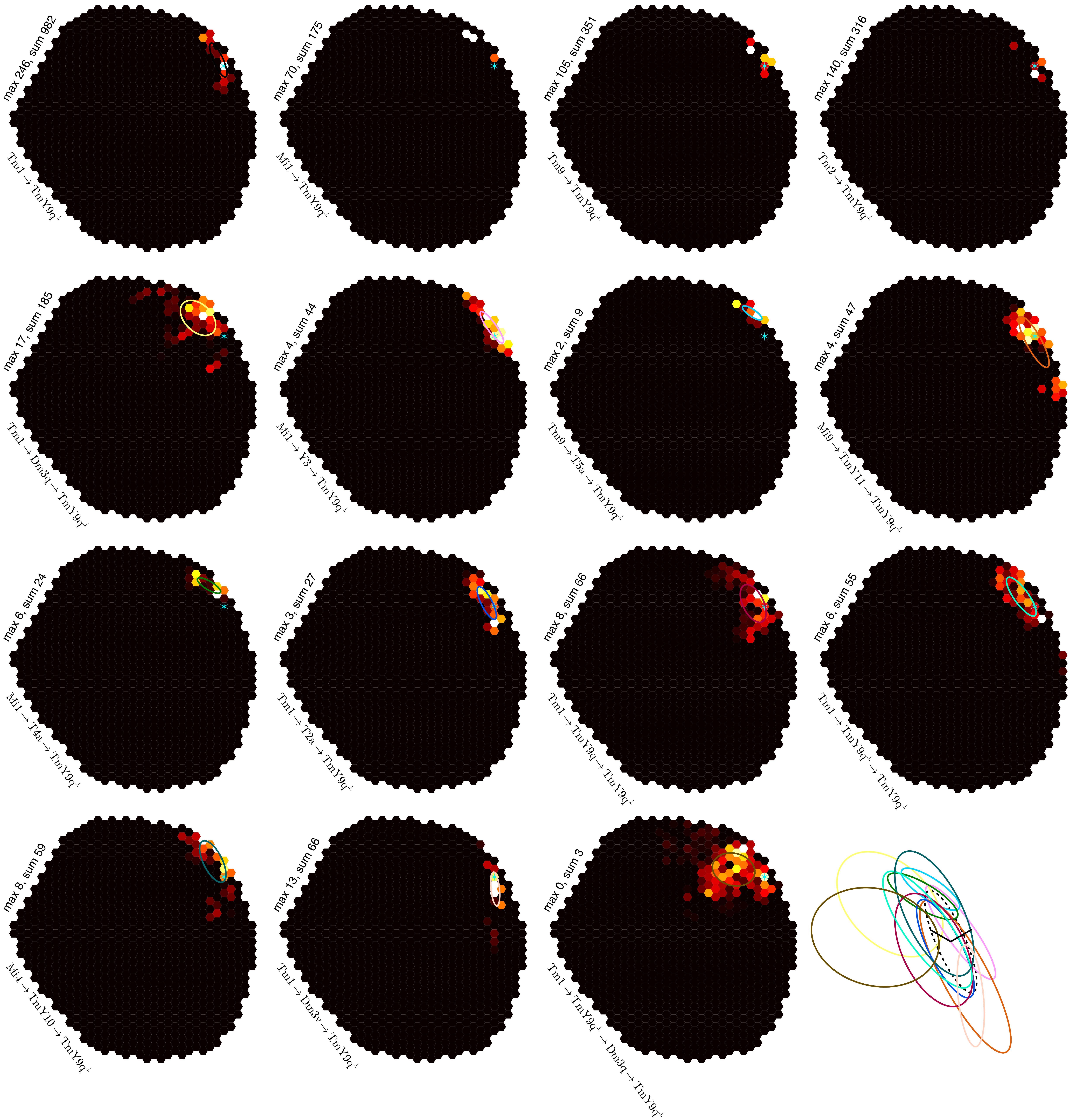
max 246, sum 982
Tm1 → TmY9q⊥
max 70, sum 175
Mi1 → TmY9q⊥
max 105, sum 351
Tm9 → TmY9q⊥
max 140, sum 316
Tm2 → TmY9q⊥
max 17, sum 185
Tm1 → Dm3q → TmY9q⊥
max 4, sum 44
Mi1 → Y3 → TmY9q⊥
max 2, sum 9
Tm9 → T5a → TmY9q⊥
max 4, sum 47
Mi9 → TmY11 → TmY9q⊥
max 6, sum 24
Mi1 → T4a → TmY9q⊥
max 3, sum 27
Tm1 → T2a → TmY9q⊥
max 8, sum 66
Tm1 → TmY9q → TmY9q⊥
max 6, sum 55
Tm1 → TmY9q⊥ → TmY9q⊥
max 8, sum 59
Mi4 → TmY10 → TmY9q⊥
max 13, sum 66
Tm1 → Dm3v → TmY9q⊥
max 0, sum 3
Tm1 → TmY9q⊥ → Dm3q → TmY9q⊥
